# Chondrocutaneous Custom-made Graft for Upper Lateral and Alar Cartilage Nose Reconstruction: The T Graft

**DOI:** 10.1055/a-2349-9835

**Published:** 2024-08-06

**Authors:** Vania Recchi, Alberto Pau, Davide Talevi, Simone Russo, Matteo Torresetti, Giovanni Di Benedetto

**Affiliations:** 1 Department of Experimental and Clinical Medicine, Clinic of Plastic and Reconstructive Surgery, Marche Polytechnic University Medical School, Ancona, Italy; 2 Department of Clinical and Molecular Sciences, Ear, Nose, and Throat Unit, Marche Polytechnic University Medical School, Ancona, Italy

**Keywords:** nasal reconstruction, composite graft, chondrocutaneous graft, internal nasal valve

## Abstract

Upper lateral cartilage and alar cartilage nose reconstruction secondary to failed aesthetic procedure or tumor excision, surely represents a reconstructive challenge for plastic surgeons, because of the support needed and for the function of the internal nasal valve (INV).

Several scientific publications deal with internal nasal reconstructive techniques, including simple homologous or heterologous tissue grafts.

We describe a new hybrid chondrocutaneous graft used for reconstruction of the upper lateral cartilage and a portion of the alar cartilage (cephalic part), excised with the adherent nasal mucosa (in correspondence with INV), included in the tumor mass.

## Introduction

Squamous cell carcinoma (SCC) of the nasal cavity is a rare malignancy with high morbidity and mortality.

Despite this, SCC is the most common cancer of the nasal cavity. Treatment options include radiotherapy or surgical excision for early lesions, while more advanced lesions require radical surgery with adjuvant radiotherapy.

An adequate knowledge of the specific functions of particular nasal portions is mandatory to achieve a functional reconstruction. The involvement of the internal nasal valve (INV), if not adequately reconstructed, is often associated with an alteration of inspiratory/expiratory flows, secondary to INV collapse. Several reconstructive methods have been described: upper lateral Strut graft, 1 alar batten graft, 2 semilunar conchal cartilage graft, 3 and auricular chondrocutaneous composite graft. 4 Additionally, some literature reviews reveal the significant role of chondrocutaneous graft in nasal reconstruction. 5 6

This new hybrid graft provides restoration of both cartilaginous and mucosal layers, with a better interface of the graft with neighboring structures thus avoiding tissue overlapping. It also allows the minimization of any closing effect of the nasal valve caused by an excessive thickness while preserving the nose aesthetic.

## Idea

**Fig. 1:**
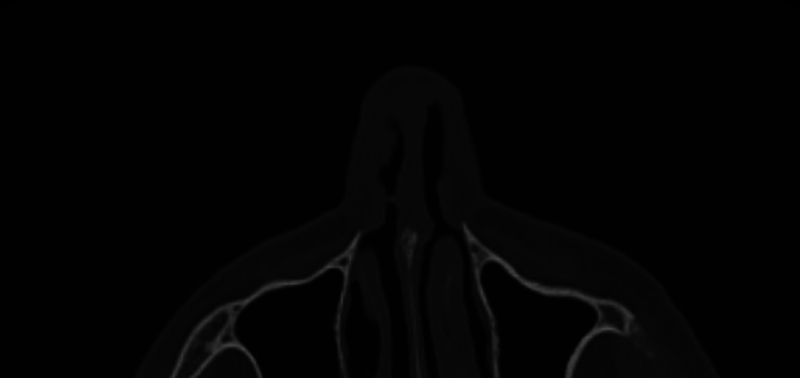
Preoperative CT: squamous cell carcinoma of the right nasal cavity with involvement of the right nasal valve.

We report the case of a 69-year-old female patient with a mucosal SCC recurrence, located in the upper part of the inner nasal valve, involving upper lateral and alar (cephalic portion) right cartilage, already subjected to previous excisions. Preoperative computed tomography (CT) excluded metastatic diffusions and the involvement of osteocartilaginous structures ( Fig. 1 ). As the tumor was located at the level of the inner nasal valve, we attempted an internal approach elevating the skin of the nasal dorsum as an open rhinoplasty. Then we excised the tumor, the defect was reconstructed, and the nasal skin was finally repositioned.

In detail, osteochondral sustaining structures of the dorsum (nasal bones and upper lateral cartilages) and alar cartilages were exposed, performing a cutaneous/subcutaneous dissection (open technique). A wide full-thickness excision of upper lateral and alar cartilage (cephalic portion) and endonasal mucosa affected by the tumor was performed. The tumor size was about 7 mm in diameter and the excision margin was about 2 to 3 mm. The “wedge” incision from the outside to the inside (cartilage to the mucosa) created a greater excision of the cartilage component than the mucosal one. The reconstruction of such important anatomical structures which have a sustaining function and a peculiar role in maintaining the patency of the inner nasal valve was attempted by creating a custom-made graft, harvested from auricular concha, in correspondence with the antelix, that we called “T graft.” This option allows for the support restoration (cartilaginous component) and reconstruction of an endonasal surface similar to normal mucosa (cutaneous component). Our goal was to achieve not only a functional result (structural preservation of inner nasal valve) but also to provide the aesthetic external aspect of the nasal pyramid.

**Fig. 2:**
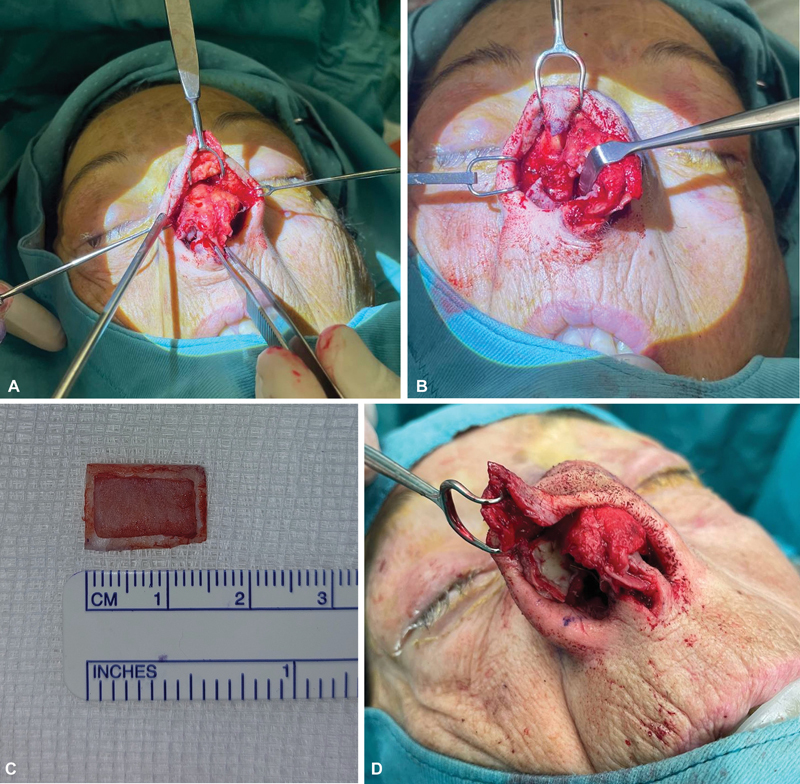
Surgical technique: exposure of the cartilaginous structures as an open rhinoplasty approach ( **A** ); upper lateral cartilage demolition after tumor excision ( **B** ); T graft with de-epithelialized skin layer ( **C** ); placement of T graft ( **D** ).

**Fig. 3:**
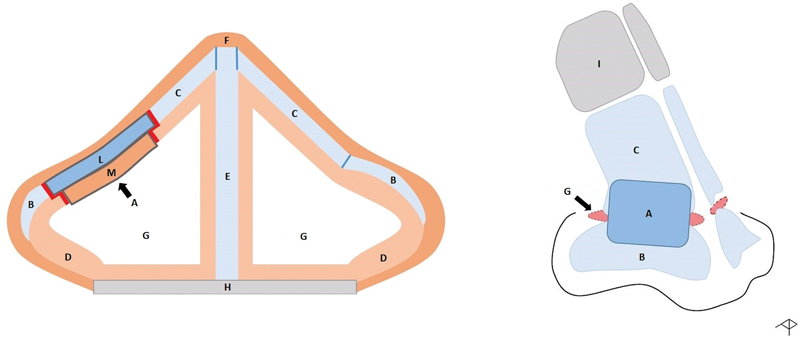
Schematic representations of T-graft connected with other nasal structures: T graft (A); alar cartilage (B); upper lateral cartilage (C); nasal mucosa (D); nasal septum (E); nasal skin (F); internal nasal valve (G); maxillae (H); nasal bone (I); auricular cartilage(L); auricular skin (M).

The graft structure was peculiar and was customized for this specific reconstruction. It had a rectangular area of 1.5 × 1 cm and a thickness of 2 to 2.5 mm, and it was composed of two layers (cartilage and skin). The skin layer was de-epithelialized around the perimeter (approximately 2–3 mm from the outer sides of the graft): a central area of the skin of approximately 1 × 0.5 cm, smaller than the cartilaginous one, was maintained ( Fig. 2A–D ). The T graft was then sutured to the remaining portion of the upper lateral cartilage, linked to the quadrangular (or septal) cartilage, and to the remaining outer portions of alar cartilage using PDS III 5–0 ( Fig. 3 ). After skin closure, Silastic splints and Merocel endonasal stents were placed. The auricular donor site was finally reconstructed with a full-thickness skin graft harvested from the inguinal region.

**Fig. 4:**
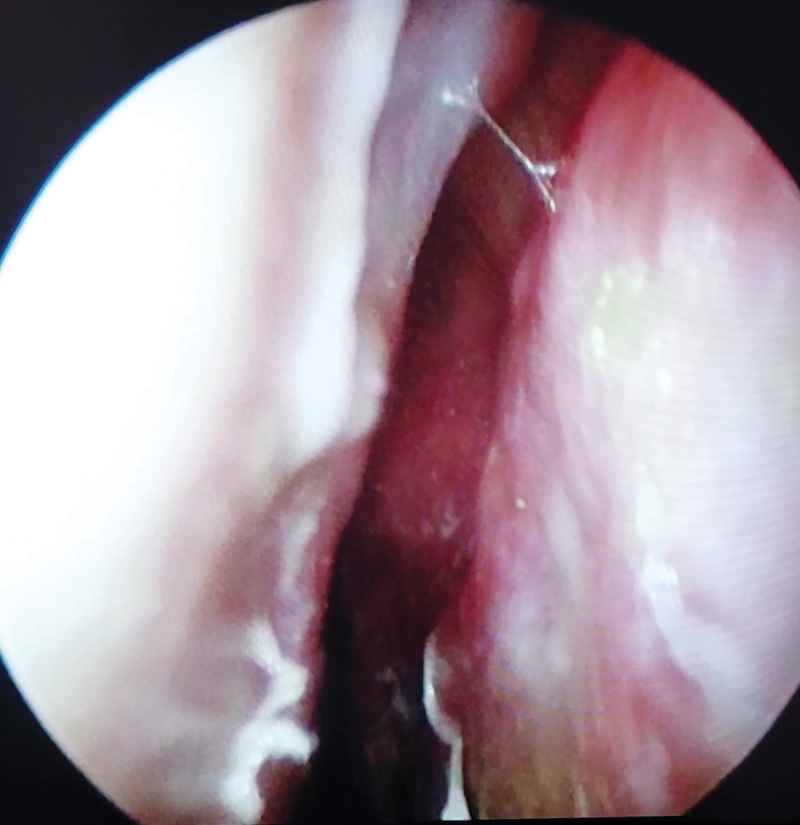
Postoperative endoscopic evaluation (6-month follow-up).

Histopathology revealed no residual SCC in the tumor bed. Six-month follow-up nasal endoscopic evaluation showed no tumor recurrence and complete engraftment of the T graft, which appeared fixed and stable with complete metaplasia of the skin layer of the T graft in correspondence with the inner nasal valve, without cartilaginous exposure ( Fig. 4 ). Based on the surgical experience of only one case, it is not possible to estimate the overall graft survival rate. However, it is assumed that the engraftment rate is determined by the vascularization of the recipient tissues, as with all other types of grafting. A recent manuscript shows several techniques useful to reduce risks of engraftment failure and a consequent estimate of the overall graft survival rate. 7 Three months postoperatively, a rhinomanometry (RYNO, Menfis BioMedica) was performed to evaluate the reconstructed inner nasal valve patency and its integrity after surgery. 8 9 Airstreams of the right nasal cavity versus the left nasal cavity were compared and evaluated. A good right nasal flow, similar to the contralateral one, was observed ( Tables 1 and 2 ).

**Table 1:** Sinusoidal analysis

Right nasal cavity	Left nasal cavity
MIP: 145 Pa	MIP: 161 Pa
MEP: −178 Pa	MEP: −158 Pa
ITV: 330 mL	ITV: 182 mL
ETV: −918 mL	ETV: −703 mL
RR: 14.1 L/min	RR: 16.4 L/min

Abbreviations: ^a^ ETV, expiratory tidal volume; ITV, inspiratory tidal volume; MEP, mean expiratory pressure; MIP, mean inspiratory pressure; RR, respiratory rate.

**Table 2:** Sigmoid analysis

Pressures	50	100	150	Pa
Right inspiratory flow	73	138	190	mL/s
Right flow increase	–	89	38	%
Right inspiratory resistance	0.68	0.62	0.79	Pa/mL s
Right expiratory resistance	0.37	0.35	0.41	Pa/mL s
Left inspiratory flow	89	215	301	mL/s
Left flow increase	–	142	40	%
Left inspiratory resistance	0.56	0.47	0.50	Pa/mL s
Left expiratory resistance	1.14	1.11	1.14	Pa/mL s
Flow ratio	0.82	0.64	0.63	–
Sum inspiratory flows	162	353	491	mL/s
Total inspiratory resistances	0.31	0.28	0.30	–
Total expiratory resistance	0.28	0.26	0.30	–

**Fig. 5:**
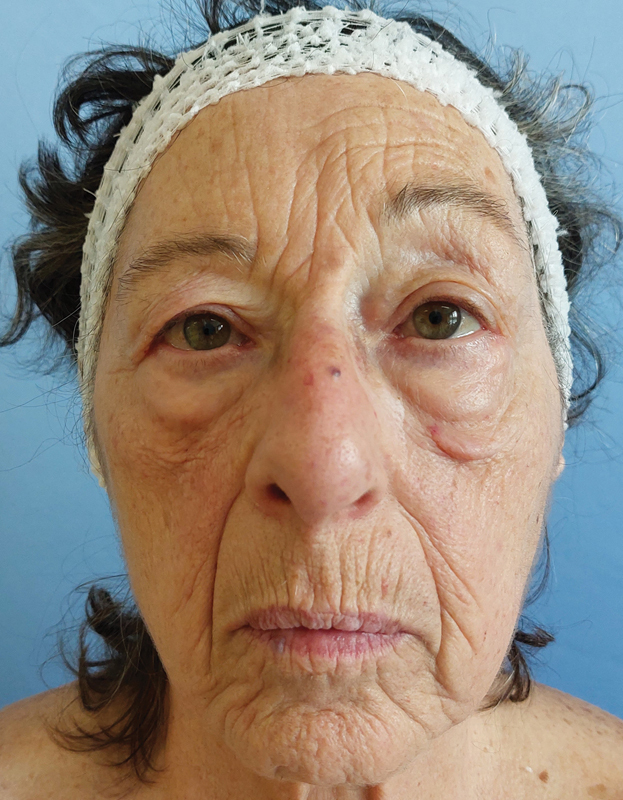
Six-month postoperative follow-up visit showing a satisfying cosmetic appearance.

The patient was also evaluated with a subjective scale (Nasal Obstruction Symptoms Evaluation Scale, 0–4 points for each parameter) after surgical reconstruction 10 11 : nasal stuffiness (1), nasal blockage or obstruction (0), trouble breathing through my nose (1), trouble sleeping (0), unable to get enough air through my nose during exercise, or exertion (1). The total detected score (3 points) showed a good subjective breath capacity. On the other hand, we obtained a satisfying cosmetic result with anatomical and functional structures preservation of the internal and external nose ( Fig. 5 ).

## Discussion

While planning the reconstructive procedures using autologous grafting after excision of a nasal tumor, which sometimes requires extensive demolition due to oncological radicality, it is important to remember some principles to prevent anatomical or functional alterations: long-term stability of nasal cartilage, donor site morbidity, graft infections, etc. In this case, the worst complication that could occur is the loss of sustenance of the upper airway, in particular the upper nasal valve. Another important issue is the aesthetic outcome. To reconstruct the nasal cartilage and the internal mucosal tissue, as physiologically as possible, we thought to apply a hybrid graft. This choice is justified by the graft double components: on one side, the cartilaginous portion with structural function, and on the other side the skin portion similar to mucosal tissue.

The composition and shape of the T-graft have innovative characteristics: the skin layer was modeled by partial de-epithelialization and exposure of an external cartilage frame, which optimized and facilitated the suturing of the cartilage component of the graft to the receiving cartilage structures (alar cartilage and upper lateral cartilage). This allowed obtaining an optimal interface, resembling a Tetris game ( Fig. 3 ), that would have been difficult to achieve if a nonselective suturing of the various layers had been performed, resulting in overlapping, potential risk of nongrafting or the occurrence of secondary complications. The residual central skin layer, clearly identifiable from the underlying, larger cartilaginous layer, was much more easily and selectively sutured to the receiving mucosa presenting areas of high vascularization. At the same time, the T graft allows to minimize any closing effect on the nasal valve caused by an excessive thickness (tissues overlapping).

The use of a skin layer provides coverage with a tissue as similar as possible to the mucosa, with complete skin metaplasia. A final consideration concerns the shape of the T-graft, which was obtained from a donor site at the antelix, with modest convexity on the skin side and modest concavity on the cartilaginous side. Due to the demolition of important and specific structural components (triangular and alar cartilage), the particular conformation of the T-graft made it possible to restore anatomical aesthetic features such as the alar crease, but also functional features such as the INV (at the involved sites). In our opinion, the T graft represents in the medium–long-term a safe, reliable, and effective method for reconstruction of the lateral superior cartilage, the alar cartilage, and the INV following oncological surgical procedures. We achieved the functional preservation of this fundamental anatomic structure and a good cosmetic outcome.
